# GntR family of regulators in *Mycobacterium smegmatis*: a sequence and structure based characterization

**DOI:** 10.1186/1471-2164-8-289

**Published:** 2007-08-23

**Authors:** Vaibhav Vindal, Katta Suma, Akash Ranjan

**Affiliations:** 1 Computational and Functional Genomics Group, Sun Centre of Excellence in Medical Bioinformatics, Centre for DNA Fingerprinting and Diagnostics, EMBnet India Node, Hyderabad 500076, India

## Abstract

**Background:**

*Mycobacterium smegmatis *is fast growing non-pathogenic mycobacteria. This organism has been widely used as a model organism to study the biology of other virulent and extremely slow growing species like *Mycobacterium tuberculosis*. Based on the homology of the N-terminal DNA binding domain, the recently sequenced genome of *M. smegmatis *has been shown to possess several putative GntR regulators. A striking characteristic feature of this family of regulators is that they possess a conserved N-terminal DNA binding domain and a diverse C-terminal domain involved in the effector binding and/or oligomerization. Since the physiological role of these regulators is critically dependent upon effector binding and operator sites, we have analysed and classified these regulators into their specific subfamilies and identified their potential binding sites.

**Results:**

The sequence analysis of *M. smegmatis *putative GntRs has revealed that FadR, HutC, MocR and the YtrA-like regulators are encoded by 45, 8, 8 and 1 genes respectively. Further out of 45 FadR-like regulators, 19 were classified into the FadR group and 26 into the VanR group. All these proteins showed similar secondary structural elements specific to their respective subfamilies except MSMEG_3959, which showed additional secondary structural elements. Using the reciprocal BLAST searches, we further identified the orthologs of these regulators in *Bacillus subtilis *and other mycobacteria. Since the expression of many regulators is auto-regulatory, we have identified potential operator sites for a number of these GntR regulators by analyzing the upstream sequences.

**Conclusion:**

This study helps in extending the annotation of *M. smegmatis *GntR proteins. It identifies the GntR regulators of *M. smegmatis *that could serve as a model for studying orthologous regulators from virulent as well as other saprophytic mycobacteria. This study also sheds some light on the nucleotide preferences in the target-motifs of GntRs thus providing important leads for initiating the experimental characterization of these proteins, construction of the gene regulatory network for these regulators and an understanding of the influence of these proteins on the physiology of the mycobacteria.

## Background

Being a fast growing, non-pathogenic mycobacteria, *Mycobacterium smegmatis *has been widely used as a model organism to study the biology of other virulent and extremely slow growing species like *M. tuberculosis*. The genome of *M. smegmatis*, as listed at the TIGR site, contains a large number of putative GntR-like regulators. These regulators play an important role in the cellular physiology. Many such regulators are involved in regulation of gene expression in response to various oxidized substrates related to either amino acid metabolism or at the branch points of various other metabolic pathways.

The GntR family of bacterial regulators is named after the *Bacillus subtilis *transcription regulator- GntR- a repressor of the gluconate operon [1]. Regulators of this family possess a conserved N-terminal domain that is involved in the DNA binding. Based on this conservation, these proteins can easily be recognized by a Conserved Domain Database (CDD) search [2]. However, the C-terminal domain, which is involved in the effector binding and/or oligomerization (E-b/O), is quite diverse and heterogeneous. As a consequence of this heterogeneity, the GntR regulators have been further classified into six subfamilies (FadR, HutC, MocR, YtrA, AraR and PlmA) [3,4]. The members of subfamilies possess conserved secondary structural features specific to their subfamily and interact with a limited number of molecules [5]. Considering these conserved secondary structural features in sequence analysis, GntR regulators are defined as a part of specific subfamily [6]. Earlier, we have characterized GntR regulators from *M. tuberculosis *[7]. In present study putative GntR regulators from *M. smegmatis *are classified into their specific subfamilies. Further, suitable orthologs of the *M. smegmatis *GntRs were also identified using reciprocal BLAST searches in *M. tuberculosis, M. avium, M. bovis, M. ulcerans, M. sp*. KMS, *M. sp*. MCS, *M. vanbaalenii *PYR-1 and *B. subtilis*. To identify the DNA targets of these regulators, we utilized the information about the nucleotide preferences for regulators of a given subfamily. All the upstream DNA sequences of the GntR coding genes were scanned to locate potential palindromes that matched the nucleotide preference criteria [5].

## Results and discussion

### Classification of the putative *M. smegmatis *GntRs into subfamilies

**Figure 1:**
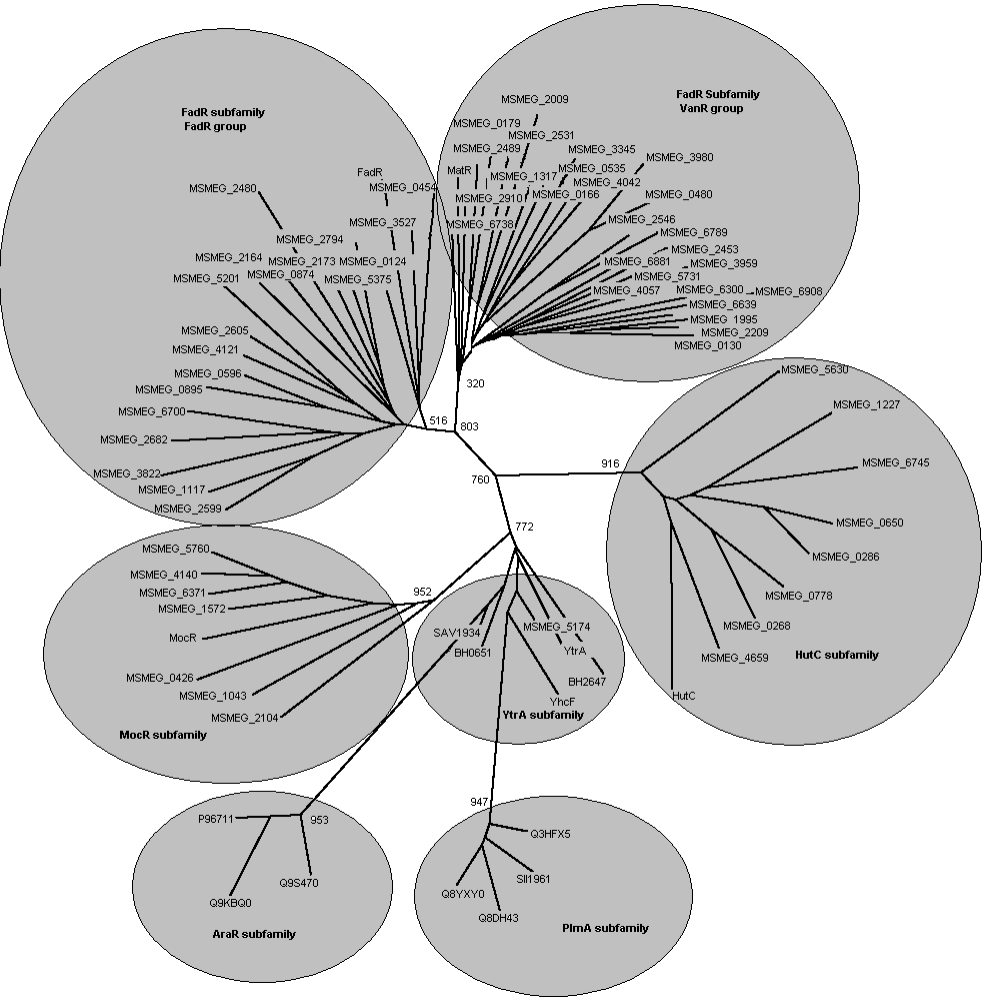
**Unrooted tree of the proteins of GntR family regulators of *M. smegmatis *including representatives of all subfamily regulators from different Bacterial Genomes with 1000 bootstrap replicates**. All the GntR regulators are clustered into six subfamilies. FadR subfamily is branched again into two groups (FadR and VanR). (Abbreviations are as indicated in Table 1 and Table 2).

Unrooted tree of the *M. smegmatis *GntRs was constructed with the classified representatives of all subfamilies (Table 1) [5]. Among all putative *M. smegmatis *GntRs two proteins (MSMEG_1043 and MSMEG_2323) were found to be identical in sequence, hence only one of them MSMEG_1043 was taken for the classification. Each branch of the constructed tree represents a subfamily. Bootstrapping, involving 1000 replicates, shows all subfamily branches clustered with high bootstrap values. FadR subfamily is divided into two groups, FadR and VanR (Figure 1).

**Table 1:** Details of GntR regulators used as representative from all subfamilies

**Subfamily**	**Organism**	**Protein ID**	**Amino acid**	**Swiss Prot ID**
FadR (FadR Group)	*Escherichia coli O157:H7*	FadR	238	P0A8V8
FadR (VanR Group)	*Rhizobium leguminosarum*	MatR	222	Q9JP74
MocR	*Rhizobium meliloti*	MocR	493	P49309
HutC	*Pseudomonas putida*	HutC	248	P22773
YtrA	*Bacillus halodurans*	BH0651	123	Q9KF35
	*Bacillus halodurans*	BH2647	123	Q9K9J9
	*Staphylococcus aureus*	SAV1934	126	Q99SV4
	*Bacillus subtilis*	YhcF	121	P54590
	*Bacillus subtilis*	YtrA	130	O34712
AraR	*Bacillus subtilis*	P96711	362	P96711
	*Bacillus halodurans*	Q9KBQ0	375	Q9KBQ0
	*Bacillus stearothermophilus*	Q9S470	364	Q9S470
PlmA	*Synechocystis sp. strain PCC 6803*	sll1961	388	P73804
	*Anabaena sp. strain PCC 7120*	Q8YXY0	328	Q8YXY0
	*Synechococcus elongatus*	Q8DH43	367	Q8DH43
	*Trichodesmium erythraeum IMS101*	Q3HFX5	327	Q3HFX5

### FadR-like proteins of *M. smegmatis*

**Figure 2:**
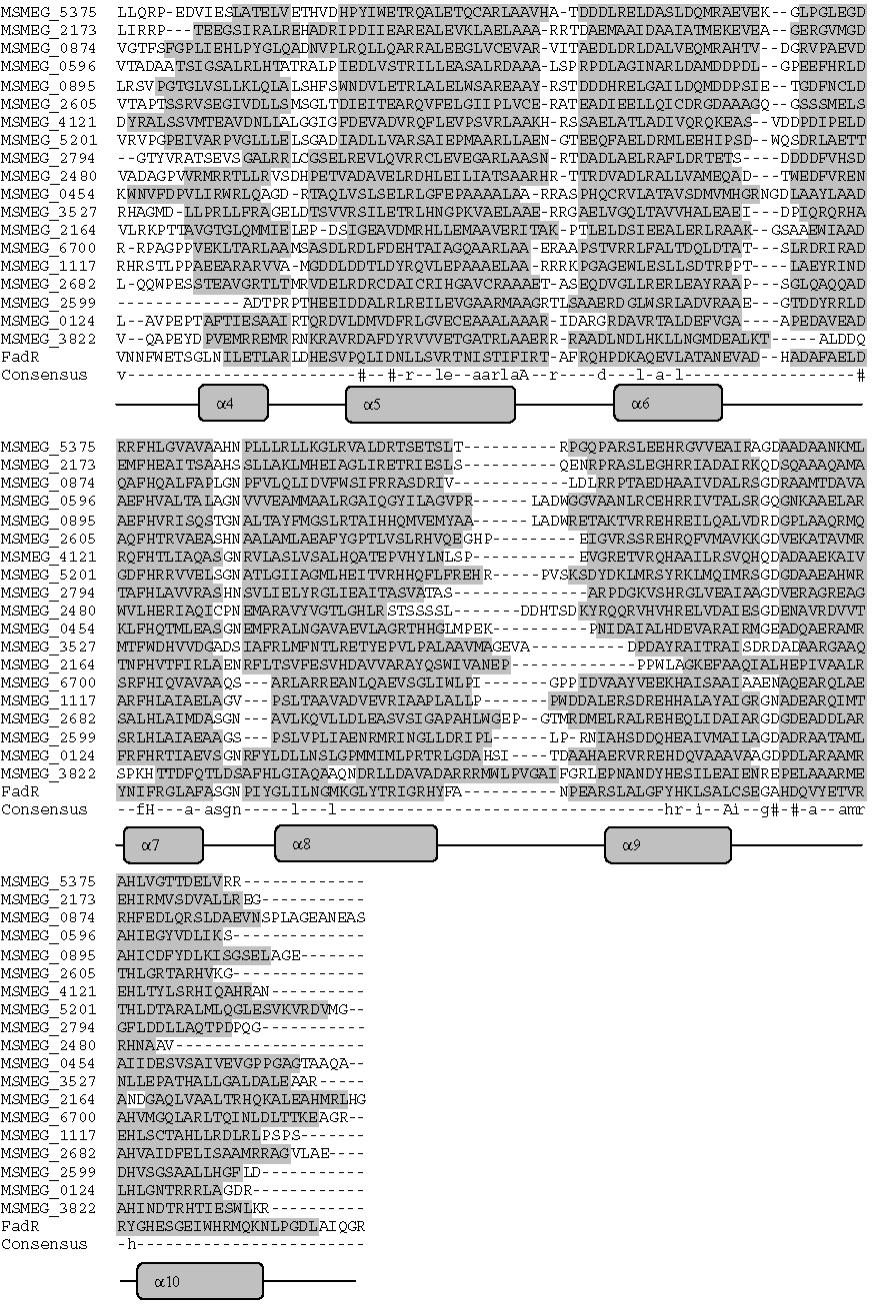
**Structure based sequence analysis of *M. smegmatis *GntR-like regulators by the multiple sequence alignment of the C-terminal domains of GntR regulators belonging to FadR Subfamily (FadR group)**. Abbreviations are as indicated in Table 1. Consensus sequence from the multiple sequence alignment has been drawn. High and low consensus levels were fixed arbitrarily at 80% and 40% of identity and are represented respectively by the capital and lowercase letters. Consensus symbol ! used for anyone of IV; $ is anyone of LM; % is anyone of FY; # is anyone of NDQEBZ. In graphical representation α-helix region and β-sheet regions are highlighted with light and dark gray background.

**Figure 3:**
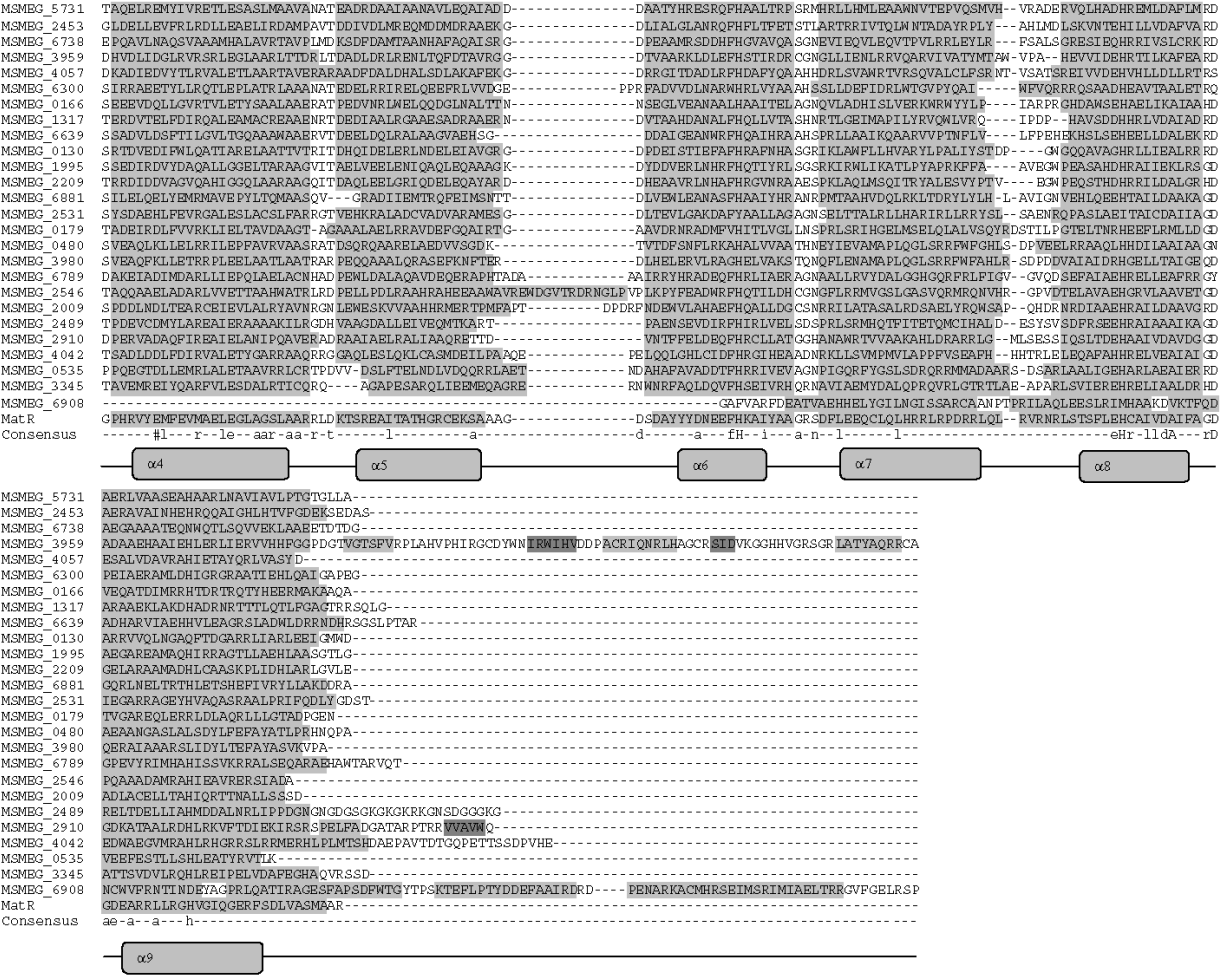
**Structure based sequence analysis of *M. smegmatis *GntR-like regulators by the multiple sequence alignment of C-terminal domains of GntR regulators belonging to FadR Subfamily (VanR group)**. Abbreviations are as indicated in Table 1. Consensus sequence from the multiple sequence alignment has been drawn. High and low consensus levels were fixed arbitrarily at 80% and 40% of identity and are represented respectively by the capital and lowercase letters. Consensus symbol ! used for anyone of IV; $ is anyone of LM; % is anyone of FY; # is anyone of NDQEBZ. In graphical representation α-helix region and β-sheet regions are highlighted with light and dark gray background.

Of all the putative GntRs, 45 proteins were classified as the FadR-like regulators. These subfamily members are further classified into two groups FadR and VanR where the C-terminal effector binding and/or oligomerization domain length is about 170 and 150 amino acid residues respectively comprising all α-helices [5]. Among all FadR-like regulators, 19 regulators were clustered as members of the FadR group while 26 for the VanR group (Table 2). To study secondary structural features both the group members were dealt with separately. C-terminal domain of all the members of FadR group were predicted with seven α-helices except MSMEG_2599. All the regulators showed distinguishable predicted secondary structural features specific to this subfamily (Figure 2 and Figure 3) [5]. Secondary structural patterns of the regulator MSMEG_3959 revealed an extra secondary structural element, which could be significant in studying protein family evolution. FadR-like regulators are known to be involved in the regulation of gene expression in response to oxidized substrates related to either amino acid metabolism or at the branch point in various metabolic pathways such as glycolate [8], pyruvate [9], lactate [10], malonate [11] or gluconate [12]. One of FadR-like classified transcriptional regulator MSMEG_6700 is known to be involved in the regulation of piperidine and pyrrolidine metabolism [13]. These results provide a starting point for a detailed biochemical and genetic characterization of *M. smegmatis *FadR-like regulators.

**Table 2:** List of Classified *M. smegmatis *GntR regulators

**Gene**	**Subfamily**	**Amino acid**	**Gene**	**Subfamily**	**Amino acid**
MSMEG_0124	FadR	227	MSMEG_2546	FadR	239
MSMEG_0130	FadR	230	MSMEG_2599	FadR	224
MSMEG_0166	FadR	242	MSMEG_2605	FadR	255
MSMEG_0179	FadR	223	MSMEG_2682	FadR	262
MSMEG_0268	HutC	292	MSMEG_2794	FadR	225
MSMEG_0286	HutC	228	MSMEG_2910	FadR	235
MSMEG_0426	MocR	469	MSMEG_3345	FadR	258
MSMEG_0454	FadR	245	MSMEG_3822	FadR	267
MSMEG_0480	FadR	219	MSMEG_3527	FadR	240
MSMEG_0535	FadR	212	MSMEG_3959	FadR	290
MSMEG_0596	FadR	228	MSMEG_3980	FadR	214
MSMEG_0650	HutC	244	MSMEG_4042	FadR	252
MSMEG_0778	HutC	246	MSMEG_4057	FadR	221
MSMEG_0874	FadR	234	MSMEG_4121	FadR	229
MSMEG_0895	FadR	247	MSMEG_4140	MocR	508
MSMEG_2323	MocR	534	MSMEG_4659	HutC	245
MSMEG_1117	FadR	239	MSMEG_5174	YtrA	121
MSMEG_1227	HutC	274	MSMEG_5201	FadR	254
MSMEG_1317	FadR	229	MSMEG_5375	FadR	230
MSMEG_1572	MocR	470	MSMEG_5630	HutC	245
MSMEG_1995	FadR	241	MSMEG_5731	FadR	240
MSMEG_2009	FadR	226	MSMEG_5760	MocR	463
MSMEG_2104	MocR	449	MSMEG_6300	FadR	224
MSMEG_2164	FadR	262	MSMEG_6371	MocR	488
MSMEG_2173	FadR	230	MSMEG_6639	FadR	222
MSMEG_2209	FadR	222	MSMEG_6700	FadR	245
MSMEG_1043	MocR	534	MSMEG_6738	FadR	227
MSMEG_2453	FadR	244	MSMEG_6745	HutC	247
MSMEG_2480	FadR	246	MSMEG_6789	FadR	246
MSMEG_2489	FadR	240	MSMEG_6881	FadR	209
MSMEG_2531	FadR	253	MSMEG_6908	FadR	221

### HutC-like proteins of *M. smegmatis*

**Figure 4:**
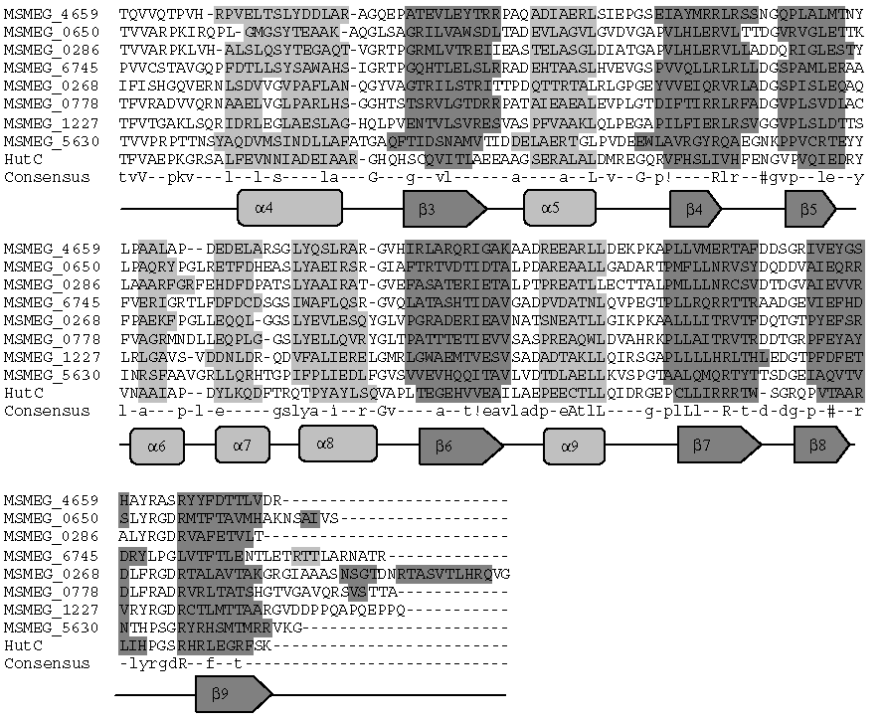
**Structure based sequence analysis of *M. smegmatis *GntR-like regulators by the multiple sequence alignment of C-terminal domains of GntR regulators belonging to the HutC Subfamily**. Abbreviations are as indicated in Table 1. Consensus sequence from the multiple sequence alignment has been drawn. High and low consensus levels were fixed arbitrarily at 80% and 40% of identity and are represented respectively by the capital and lowercase letters. Consensus symbol ! used for anyone of IV; $ is anyone of LM; % is anyone of FY; # is anyone of NDQEBZ. In graphical representation α-helix region and β-sheet regions are highlighted with light and dark gray background.

Contrary to the FadR-like regulators, the regulators of this subfamily consist of both α-helices and β-sheet structures in the C-terminal domain. We identified eight GntRs as members of this subfamily (Table 2). All these members showed distinguishable predicted secondary structural features specific to this subfamily (Figure 4) [5]. These regulators are known to acquire the same protein fold as *Escherichia coli *UbiC; hence it is also named as UbiC transcription regulator-associated (UTRA) domain [14]. This effector-binding domain responds to various ligands like histidine (HutC) [15], long chain fatty acids [16], trehalose 6-phosphate [17] or alkylphosphonate [18]. A range of known ligands, specific to many HutC-like regulators, will help in characterizing the classified *M. smegmatis *regulators.

### MocR-like protein of *M. smegmatis*

**Figure 5:**
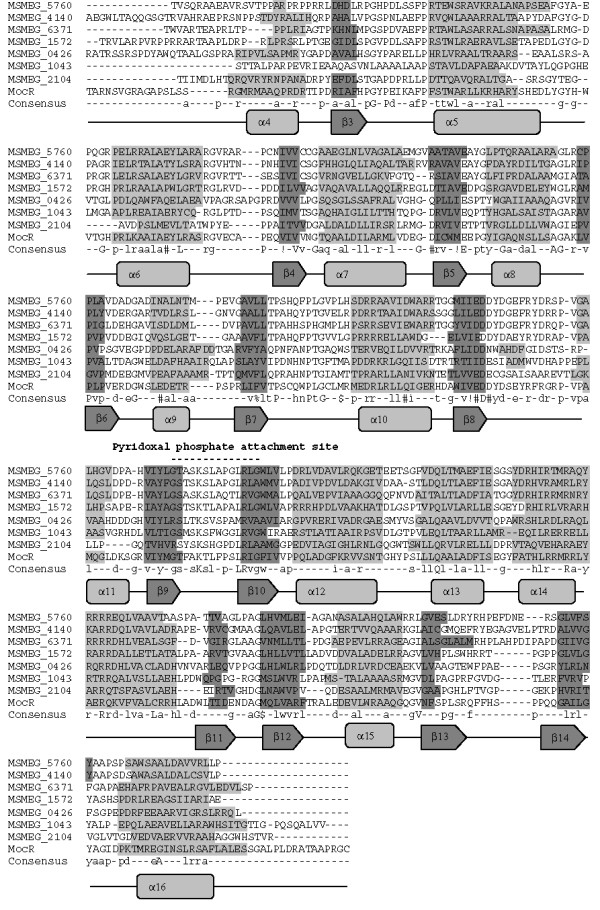
**Structure based sequence analysis of *M. smegmatis *GntR-like regulators by the multiple sequence alignment of C-terminal domains of GntR regulators belonging to the MocR Subfamily**. Abbreviations are as indicated in Table 1. Consensus sequence from the multiple sequence alignment has been drawn. High and low consensus levels were fixed arbitrarily at 80% and 40% of identity and are represented respectively by the capital and lowercase letters. Consensus symbol ! used for anyone of IV; $ is anyone of LM; % is anyone of FY; # is anyone of NDQEBZ. In graphical representation α-helix region and β-sheet regions are highlighted with light and dark gray background.

Among all the putative GntR regulators, eight were classified as members of the MocR subfamily (Table 2). All the eight regulators showed distinguishable predicted secondary structural features specific to this subfamily (Figure 5) [5]. MocR-like regulators show homology to the class I aminotransferase proteins [19], which requires pyridoxal 5'-phosphate (PLP) as a co-factor. All MocR-like regulators exhibit a PLP attachment site with a conserved lysine residue, which is also evident in the classified MocR-like regulators (Figure 5). It would thus be interesting to study the role of pyridoxal phosphate regulation in the classified regulators. [20].

### YtrA-like protein of *M. smegmatis*

**Figure 6:**
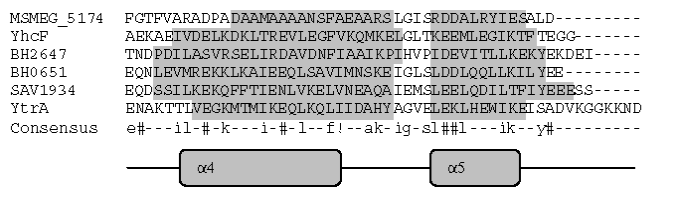
**Structure based sequence analysis of *M. smegmatis *GntR-like regulators by the multiple sequence alignment of the C-terminal domains of GntR regulators belonging to YtrA Subfamily**. Abbreviations are as indicated in Table 1. Consensus sequence from the multiple sequence alignment has been drawn. High and low consensus levels were fixed arbitrarily at 80% and 40% of identity and are represented respectively by the capital and lowercase letters. Consensus symbol ! used for anyone of IV; $ is anyone of LM; % is anyone of FY; # is anyone of NDQEBZ. In graphical representation α-helix region and β-sheet regions are highlighted with light and dark gray background.

The YtrA subfamily is the least represented GntR-like regulator in the bacterial genomes. Among all *M. smegmatis *GntR regulators, only one regulator MSMEG_5174, showed the signatures of the YtrA subfamily member (Table 2, Figure 6). YtrA possesses a reduced C-terminal domain with only two α-helices. The average length of the putative effector binding and/or oligomerization domain is about 50 amino acids [5]. YtrA from *B. subtilis *is an experimentally explored regulator, which is part of a large self-regulated operon. This operon consists of genes encoding the ATP binding cassette (ABC) transport systems in addition to the YtrA [21]. It would be interesting to study further, whether MSMEG_5174 has any role in modulating such an operon.

### Operator/binding site analysis

We have tabulated a list of potential operator sites near the perfect palindrome sequence with conserved residues, which are found to be specific for most of the subfamily members (Table 3) [5]. We did not find an operator sequence in the upstream sequences of all the remaining regulators. All the predicted sites were found to be in the upstream region from the translation start site except MSMEG_2599. Identification of these sites is an important step to understand the GntR associated regulon or the gene regulatory network in the genome [22-25].

**Table 3:** List of predicted potential operator sites

**Subfamily**	**Regulator**	**Potential Operator sequence**
**FadR**	MSMEG_0124	--CCACT**GT**TCA**AC**G**A**GCG---
	MSMEG_0179	-AAGA**T**C**GT**CCG**AC**A**A**TT----
	MSMEG_0454	--CAA**T**C**GT**CAT**AC**G**A**TTG---
	MSMEG_0596	--GTG**T**G**GT**CAG**AC**C**A**CAC---
	MSMEG_0895	-----**T**C**GT**GGG**AC**G**A**------
	MSMEG_2164	-----CC**GT**TGA**AC**GG------
	MSMEG_2480	---ACCG**GT**GGC**AC**C**A**GGGT--
	MSMEG_2599	----ACC**GT**GGG**AC**GGT-----
	MSMEG_2682	-----TG**G**CAAG**AC**C**A**------
	MSMEG_2910	CCTTGAT**GT**CCC**AC**A**A**CG----
	MSMEG_3527	-----**T**G**GT**AAG**AC**C**A**------
	MSMEG_3822	-----**T**T**GT**TACT**C**A**A**------
	MSMEG_3959	--TTGCC**G**CGCG**AC**A**A**------
	MSMEG_3980	-----**T**G**GT**GAT**AC**ACCA----
	MSMEG_4057	----T**T**C**GT**GTC**AC**A**A**GTCGAA
	MSMEG_6789	----T**T**T**GT**GTC**AC**A**A**A-----
**HutC**	MSMEG_0268	-----ACC**GT**C**TA**C**A**TCGT---
	MSMEG_0650	------TG**GT**C**TA**T**AC**CA----
**YtrA**	MSMEG_5174	---**G**CCA**T**CATG**TA**GTG**C**----

Preferred nucleotides in potential operator sites are printed in bold

### Ortholog prediction

We have found a number of *M. smegmatis *GntR regulators that are orthologs of proteins from the other species of mycobacteria and *B. subtilis *(Table 4). As orthologs typically share the same function, these regulators could serve as a model to study homologues from the other species of mycobacteria. These characterized orthologs may provide clues for initiating detailed biochemical characterization of *M. smegmatis *proteins. Many putative orthologs were experimentally known like Rv0165c that is involved in regulation of *mce1 *operon [6]; GntR, a transcriptional repressor of gluconate operon [12]; YcbG, involved in utilization of D-glucarate and D-galactarate [26]; YcnF, involved in utilization of gamma-aminobutyrate [27]. However, we did not find the orthologs for all *M. smegmatis *GntRs in other pathogenic species.

**Table 4:** Orthologs of *M. smegmatis *GntR-like regulators in other bacterial species

***M.smeg***	***M.tub***	***M.aviump***	***M.bov***	***M.van***	***M.spMCS***	***M.spKMS***	***M.ulc***	***B.sub***
MSMEG_0130	Rv0165c	MAP3599c	Mb0170c	Mvan_0130	Mmcs_0114	Mkms_0123	MUL_1058	-
MSMEG_0179	-	-	-	-	-	-	MUL_1833	-
MSMEG_0268	-	-	-	Mvan_5574	Mmcs_0189	Mkms_0198	-	-
MSMEG_0286	-	-	-	Mvan_0056	-	-	-	-
MSMEG_0454	-	-	-	Mvan_5910	-	Mkms_5416	-	-
MSMEG_0535	-	-	-	-	-	-	-	GntR
MSMEG_0596	-	-	-	-	-	Mkms_4471	-	-
MSMEG_1043	-	-	-	Mvan_2084	-	Mkms_1901	-	-
MSMEG_1227	-	MAP1105	-	-	-	-	-	-
MSMEG_1317	-	-	-	Mvan_3051	-	-	-	-
MSMEG_2104	-	MAP1267	-	-	-	-	MUL_1552	-
MSMEG_2173	-	-	-	Mvan_0294	-	-	-	YcbG
MSMEG_2209	-	MAP2404c	-	Mvan_1978	-	Mkms_1807	MUL_3894	-
MSMEG_2599	-	-	-	Mvan_2282	-	Mkms_2107	-	-
MSMEG_2794	-	-	-	Mvan_0952	-	Mkms_0349	MUL_1381	-
MSMEG_3527	Rv0586	-	Mb0601	Mvan_2942	-	Mkms_2771	MUL_4564	-
MSMEG_3822	-	-	-	Mvan_0606	-	Mkms_0519	-	-
MSMEG_4057	-	-	-	-	-	-	-	YdhC
MSMEG_4140	-	-	-	-	-	-	-	YcnF
MSMEG_4659	Rv0792c	MAP0628c	Mb0816c	Mvan_4015	-	-	MUL_0525	YvoA
MSMEG_5174	Rv1152	MAP2632c	Mb1183	Mvan_4569	-	-	MUL_0993	YtrA
MSMEG_5201	Rv3060c	MAP2347	Mb3086c	Mvan_4590	-	Mkms_4157	MUL_3832	-
MSMEG_5630	-	MAP3505c	-	Mvan_4965	-	Mkms_4496	MUL_4818	-
MSMEG_5731	-	-	-	Mvan_0931	-	Mkms_4957	-	-
MSMEG_6371	-	-	-	Mvan_5625	-	Mkms_5086	-	YhdI
MSMEG_6700	-	-	-	Mvan_1846	-	-	-	-
MSMEG_6908	Rv0043c	MAP0053c	Mb0044c	Mvan_6046	-	Mkms_5471	MUL_0061	-

'-' Represents, corresponding orthologs are not present in the genome. *M.smeg – M. smegmatis; M.tub – M. tuberculosis; M.aviump. – M. avium para.; M.bov – M. bovis; M.van *– *M. vanbaalenii *PYR-1; *M.sp*MCS – *M. sp*. MCS;*M.sp*KMS – *M. sp*. KMS; *M.ulc – M. ulcerans; B.sub – B. subtilis*.

Our results help in extending the annotation of GntRs encoded in the *M. smegmatis *genome. We have classified putative *M. smegmatis *GntRs into four subfamilies. Though in the present study, we have made an attempt to explore *M. smegmatis *GntR regulators, this approach could also be effectively employed to extend the GntR family classification in other bacterial species as well.

## Conclusion

This analysis has shown that *M. smegmatis *is equipped with large number of GntR-like regulators, belonging to four subfamilies. It further suggests that the GntR regulatory repertoires of *M. smegmatis *are far more complex than in *M. tuberculosis*. Indeed, additional GntR regulators possibly control a subset of genes required for adapting to a range of environmental conditions. One of the FadR-like regulators shows additional secondary structural elements, suggesting a possible origin of a new group within the FadR subfamily. Identified orthologs from *M. smegmatis *could serve as a model to decipher molecular regulation events taking place in the pathogenic mycobacteria. Potential operator sites were also identified based on the nucleotide recognition preferences of GntR-like regulators.

## Methods

### Selection of GntR-like Members

The sequences of *M. smegmatis MC2 *were downloaded from the Institute for Genomic Research Comprehensive Microbial Resource [28]. Apart from classified GntR regulators or proteins annotated as GntR-like regulator, other putative GntRs from *M. smegmatis *proteome were selected using GntR Pfam profile [29]. Among all predicted GntRs one protein (MSMEG_3400) was discarded for this study because of its unusual size (741 amino acid) and its annotation as glutamyl-tRNA(Gln) amidotransferase subunit A. Rest of the GntR regulators were retrieved from the SWISS-PROT/TrEMBL sequence database as per their Swiss-Prot ID (Table 1). Additionally published and annotated genome sequences of *M. tuberculosis*, *M. avium subsp. paratuberculosis*, *M. bovis, M. ulcerans, M. sp *KMS, *M. sp. MCS, M. vanbaalenii *PYR-1 and *Bacillus subtilis *were downloaded from the NCBI ftp site [30].

### Secondary structure prediction

The secondary structural features of all bacterial GntR regulators including the *M. smegmatis *GntRs were analyzed (Table 1 and Table 2). Secondary structure predictions were made using Jpred [31], SsPro [32] and 3DPSSM [33]. A consensus of all the secondary structure predictions was considered for a better validity.

### Multiple sequence alignments and Phylogenetic tree construction

Multiple sequence alignment was generated with MULTIALIN [34]. Distances between aligned proteins were computed with the PROTDIST program using the Dayhoff PAM matrix [35]. The FITCH program estimated phylogenies from distances in the matrix data using the Fitch-Margoliash algorithm [36]. The phylogenetic tree was drawn using the TREEVIEW program with incorporation of bootstrap values that were obtained involving 1000 replicates [37]. PROTDIST and FITCH programs are included in the PHYLIP package developed by Felsenstein [38].

### Operator site analysis

To study the upstream region of GntR-like regulators, we considered sequences from 400 bases upstream to 50 bases downstream from the translation start site. As many GntR regulators are reported to recognize palindromes and also exhibit nucleotide recognition preferences among the same subfamily [5], we utilised these clues to scan the upstream sequences.

### Reciprocal BLAST

Reciprocal BLAST hits are frequently utilized to identify the orthologs in two species [39,40]. In this method we searched for the best reciprocal BLAST hit for *M. smegmatis *GntR proteins with *M. tuberculosis, M. avium, M. bovis, Mycobacterium ulcerans, Mycobacterium sp *KMS, *Mycobacterium sp*. MCS, *Mycobacterium vanbaalenii *PYR-1 and *B. subtilis*.

## Abbreviations

*M. tuberculosis *– *Mycobacterium tuberculosis*

M. bovis – Mycobacterium bovis

M. avium para. – Mycobacterium avium subsp. paratuberculosis

M. smegmatis – Mycobacterium smegmatis

M. ulcerans – Mycobacterium ulcerans

*M. sp *KMS – *Mycobacterium sp*. KMS

*M. sp*. MCS – *Mycobacterium sp*. MCS

*M. vanbaalenii *PYR-1 – *Mycobacterium vanbaalenii *PYR-1.

## Authors' contributions

VV carried out the operator site prediction, subfamily data analysis, ortholog search and drafted the manuscript. KS participated in the multiple sequence alignment and structure based manual adjustment. AR participated in the study design and coordination. All authors read and approved the final manuscript.
